# Supplementary material for: Targeted enhancement of the therapeutic window of L19-TNF by transient and selective inhibition of RIPK1-signaling cascade
Source: Oncotarget. 2019 Nov 19;10(62):6678–90. doi: 10.18632/oncotarget.27320 (PMC6877107; doi:10.18632/oncotarget.27320)
Supplement: Supplementary file 1 [file oncotarget-10-6678-s001.pdf]

Targeted enhancement of the therapeutic window of L19-TNF by transient and selective inhibition of RIPK1-signaling cascade

SUPPLEMENTARY MATERIALS

PROTEIN PRODUCTION AND PURIFICATION

The L19-mTNF fusion protein (sequence reported below) was cloned into the mammalian expression vector pcDNA3.1(+) (Invitrogen) using a strategy similar to the one described before [1]. The protein was expressed in CHO-S cells (Invitrogen) by transient gene expression. Briefly, CHO cells in suspension were first counted and resuspended in fresh ProCHO medium to a final cell concentration of 4 × 10^6 cells/mL. 0.75 µg DNA/million cell and 2.5 µg PEI/million cells were added carefully to the cells. Cells were incubated in a shaker at 31 °C × 150 rpm for 6 days. After incubation the suspension was centrifuged at 4 °C × 4000 rpm for 30 minutes (JA-10 rotor) using AVANTI J-26S XP centrifuge (Beckman Coulter). Supernatant was filtered with 0.45µM filters (Nalgene) and incubated for 2h at room temperature with Protein A agarose beads resin (Sino Biological) before loading onto the PD-10 column. The column was thereafter washed with 200 mL of Buffer A (100 mM NaCl, 0.5 mM EDTA, 0.1% Tween 20 in PBS) and then with 200 mL Buffer B (500 mM NaCl, 0.5 mM EDTA in PBS). The antibody product was eluted using 10-15 mL 0.1 M glycine at pH = 3 and fractions of 1 mL were collected. OD at an absorbance of 280 nm (OD_{280}) was measured and fractions containing protein (OD_{280} > 0.1 mg/mL) were pooled and loaded on SpectraPor dialysis membrane MW 3500 (Spectrum laboratories) and dialyzed in PBS o/n at 4 °C. After dialysis, L19-mTNF was characterized by SDS-PAGE, size exclusion chromatography, surface plasmon resonance and mass spectrometry.

PROTEIN SEQUENCE OF L19-MTNF

EVQLLESGGGLVQPGGSLRLSCAASGFTFSSFS MSWVRQAPGKGLEWVSSISGSSGTTYYADSVKGRF TISRDNSKNTLYLQMNSLRAEDTAVYYCAKPFPYFD YWGQGTLVTVSSGDGSSGGSGGASEIVLTQSPGTLS LSPGERATLSCRASQSVSSSFLAWYQQKPGQAPRLL IYYASSRATGIPDRFSGSGSGTDFTLTISRLEPEDFAV YYCQQTGRIPPTFGQGTKVEIKSSSSGSSSSGSSSSG LRSSSQNSSDKPVAHVVANHQVEEQLEWLSQRANA LLANGMDLKDNQLVVPADGLYLVYSQVLFKGQGC PDYVLLTHTVSRFAISYQEKVNLLSAVKSPCPKDTPE GAELKPWYEPIYLGGVFQLEKGDQLSAEVNLPKYL DFAESGQVYFGVIAL

PROTEIN SEQUENCE OF L19-HTNF

EVQLLESGGGLVQPGGSLRLSCAASGFTFSSF SMSWVRQAPGKGLEWVSSISGSSGTTYYADSVKG RFTISRDNSKNTLYLQMNSLRAEDTAVYYCAKPFP YFDYWGQGTLVTVSSGDGSSGGSGGASEIVLTQSP GTLSLSPGERATLSCRASQSVSSSFLAWYQQKPGQ APRLLIYYASSRATGIPDRFSGSGSGTDFTLTISRLEP EDFAVYYCQQTGRIPPTFGQGTKVEIKEFSSSSGSSS SGSSSSGVRSSSRTPSDKPVAHVVANPQAEGQLQW LNRRANALLANGVELRDNQLVVPSEGLYLIYSQVL FKGQGCPSTHVLLTHTISRIAVSYQTKVNLLSAIKSP CQRETPEGAEAKPWYEPIYLGGVFQLEKGDRLSAE INRPDYLDFAESGQVYFGIIAL

PROTEIN CHARACTERIZATION

SDS-PAGE

Protein samples were diluted to 0.2-0.3 mg/mL in PBS and mixed with either reducing or non-reducing 5x Loading buffer. Samples were denatured 5' at 95 °C and loaded on NuPAGE 4-12% Bis-Tris Gel (Novex™ by Life Technologies). 1x MES NuPAGE (Novex™ by Life Technologies) was used as running buffer and electrophoresis was performed at 180 V, 110 mA for 1h. Gel was rinsed with deionized water and stained in Coomassie blue for 15-20' on an orbital shaker. Staining solution was discarded and the gel was rinsed 3 times with deionized water and immerged in destaining solution (10% acetic acid/30% methanol/mQ water) for 3-12h on an orbital shaker. Destaining solution was discarded and recycled, gel was rinsed with deionized water and a picture of the gel was taken. Recipes for the 5X Loading buffer and Coomassie blue stain are as described in Supplementary Table 1 and 2.

Gel filtration analysis

100 µL of diluted sample (final concentration 0.1-0.5 mg/mL) were loaded on FPLC (Äkta, GE Healthcare) and

Supplementary Table 1: 5X Loading buffer recipe. For 5X reducing Loading buffer, add 5-10% (v/v) 2-mercaptoethanol

100mL, 5X non-red Loading Buffer	
Tris-HCl (250mM, pH 6.8)	20.8 mL
Glycerol	33.3 mL
SDS	6.6 g
Bromophenol blue	66 mg
mQ water	up to 100 mL

Supplementary Table 2: Coomassie blue recipe

1L coomassie blue	
PlusOne Coomassie PhastGel Blue R-350	2 tablets
Methanol	400 mL
Acetic Acid	100 mL
mQ water	500 mL

protein were separated by a Superdex200 Increase 10/300 GL column (GE Healthcare) previously equilibrated with 1 CV PBS, using PBS as mobile phase at a flow rate of 0.6 mL/min (column pressure limit set at 5 MPa). Proteins were detected by an UV-detector at a wavelength of 280 nm.

Mass spectrometry

Samples were diluted to about 0.1 mg/mL and LC-MS was performed on a Waters Xevo G2XS Qtof instrument (ESI-ToF-MS) coupled to a Waters Acquity UPLC H-Class System using a 2.1 × 50 mm Acquity BEH300 C4 1.7 µm column (Waters). 0.1% FA in water (solvent A) and 0.1% FA in MeCN (solvent B) were used as mobile phase at a flow rate of 0.4 mL/min. Gradient was programmed as follows: after 1.5 min isocratic with 95% solvent A, stepwise change from 95% solvent A to 95% solvent B in 4.5 min (10% increase every 0.5 min), back to 95% solvent A in 0.5 min, linearly to 95% solvent B and back to 95% solvent A in 2.25 min (last step repeated twice).

IN VITRO CYTOTOXICITY ASSAYS OF L19-HTNF ON WEHI-164 IN COMBINATION WITH RIPK1 INHIBITORS

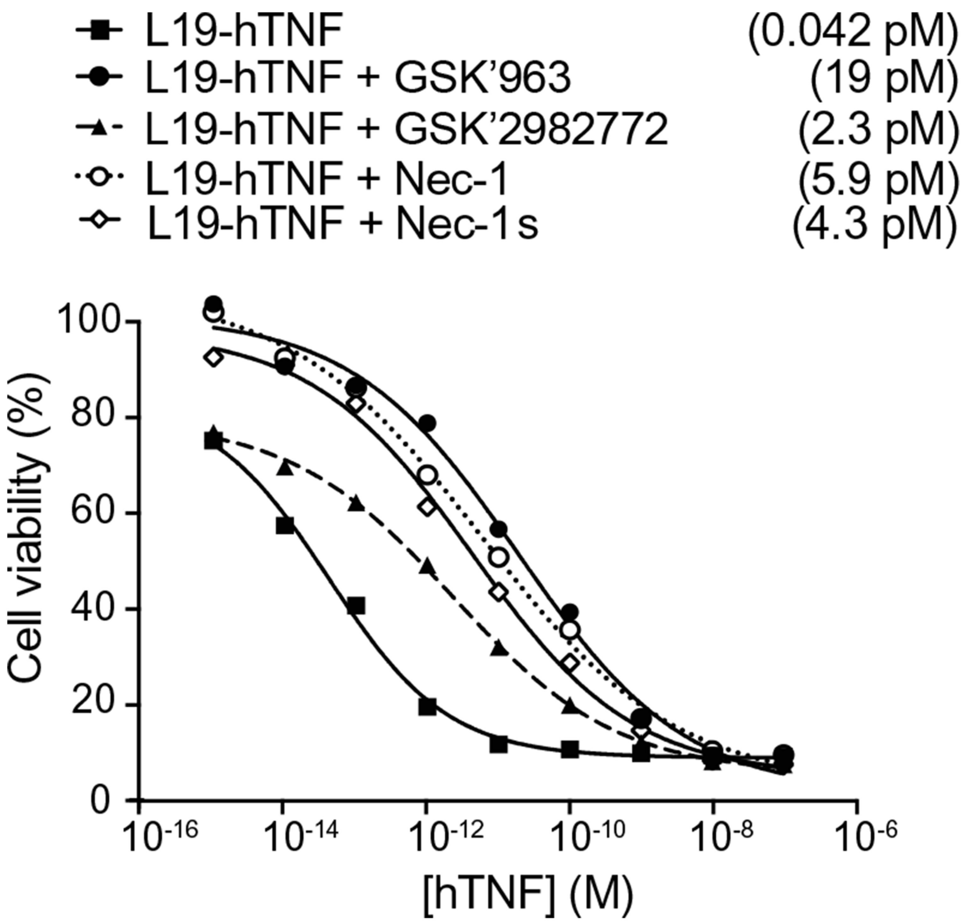

Supplementary Figure 1: *In vitro* activity of L19-hTNF alone or in combination with small molecule RIPK1 inhibitors. Dose-response curves of L19-hTNF (■ □) obtained in the presence or absence of 1μM of GSK'963 (●), GSK'2982772 (▲), Necrostatin-1 (○) or Necrostatin-1s (◊) on WEHI-164 murine fibrosarcoma**.** Each data value represents the mean of cell viability ± SD (n = 3). The potency of L19-hTNF is expressed as calculated IC50 values in brackets.

EX VIVO IMMUNOFLUORESCENCE APOPTOSIS ANALYSIS

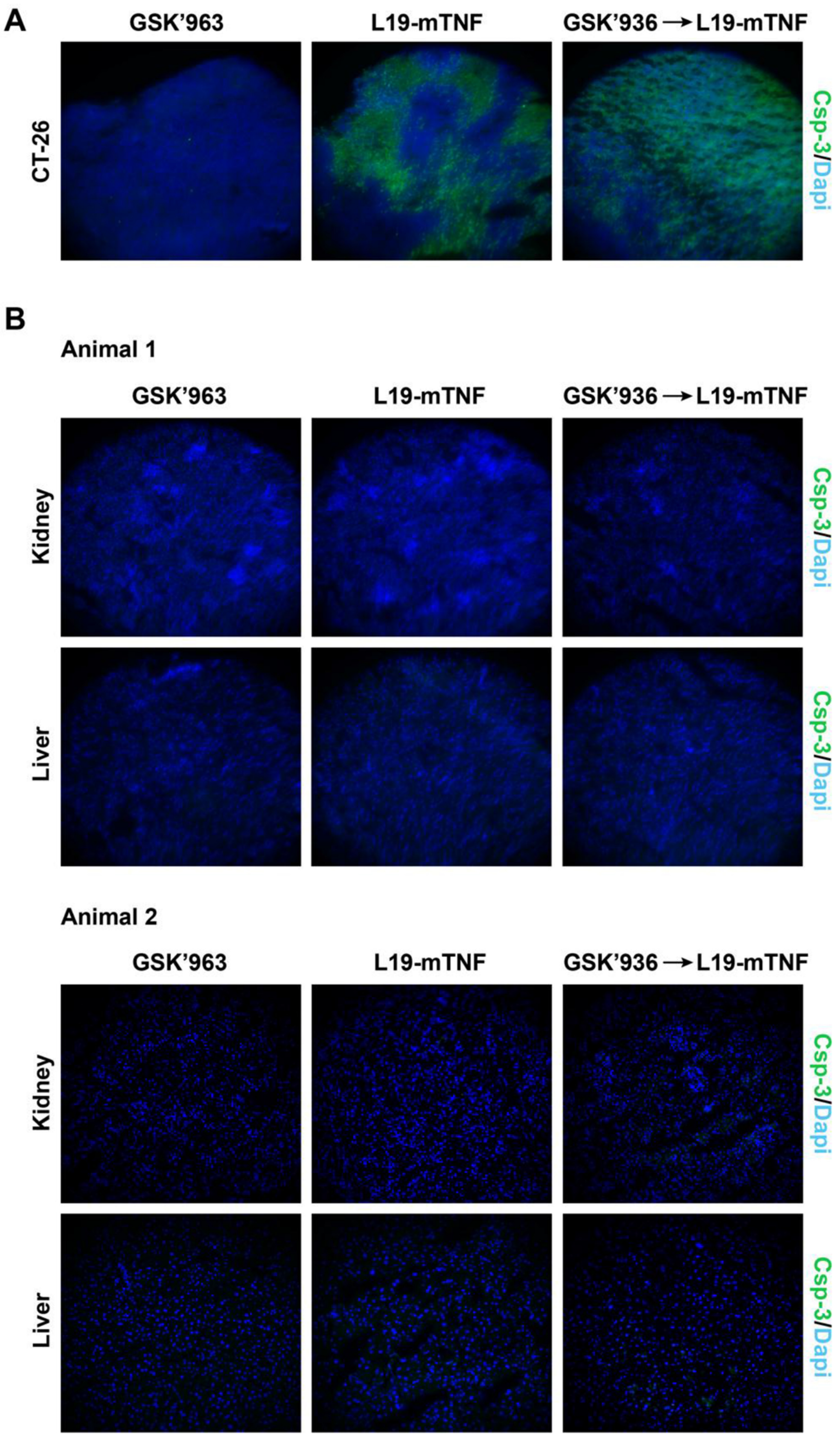

Supplementary Figure 2: Apoptosis staining after L19-mTNF treatment (250 µg/Kg) alone or in combination with GSK'963 (2 mg/Kg) in CT-26 tumor-bearing mice (n = 2). Twenty- four hours after the i.v. injections, healthy organs (kidney and liver) and tumors were excised and processed as describe before in the material and methods. Slides were stained using a rabbit anti-caspase-3 antibody (apoptotic cells; 1:200 dilution; Sigma) and detected with a goat anti-rabbit AlexaFluor488 secondary antibody (green; 1:500 dilution; Invitrogen). Nuclei were counterstained with DAPI (blue; 1:1000; Invitrogen). Pictures represent a significant region of the entire sample (20x magnification).

STATISTICAL ANALYSIS OF THERAPY EXPERIMENTS

Differences in tumor volume and body weight between therapeutic groups were compared using the two-way ANOVA analysis with Bonferroni post-test of Graphpad Prism 7 (La Jolla, CA, USA). Days are counted after tumor implantation.

Tumor Size (mg)

GSK'963 (2 mg/Kg) vs L19-mTNF (250 µg/Kg).
From day 4 to day 9 non-significant differences
day 10 $p < 0.05$
day 11 $p < 0.001$
from day 12 $p < 0.0001$

GSK'963 (2 mg/Kg) vs GSK'963 (2 mg/Kg) → L19-mTNF (250 µg/Kg).
From day 4 to day 11 non-significant differences
From day 12 $p < 0.01$

L19-mTNF (250 µg/Kg) vs GSK'963 (2 mg/Kg) → L19-mTNF (250 µg/Kg).
From day 4 to day 13 non-significant differences

GSK'963 (2 mg/Kg) vs L19-mTNF (375 µg/Kg).
day 4 non-significant differences
day 5 non-significant differences
day 6 $p < 0.05$

GSK'963 (2 mg/Kg) vs GSK'963 (2 mg/Kg) → L19-mTNF (375 µg/Kg).
From day 4 to day 9 non-significant differences
day 10 $p < 0.05$
day 11 non-significant differences
from day 12 $p < 0.001$

L19-mTNF (375 µg/Kg) vs GSK'963 (2 mg/Kg) → L19-mTNF (375 µg/Kg).
From day 4 to day 6 non-significant differences

L19-mTNF (250 µg/Kg) vs L19-mTNF (375 µg/ Kg).
From day 5 to day 6 non-significant differences

L19-mTNF (250 µg/Kg) vs GSK'963 (2 mg/Kg) → L19-mTNF (375 µg/Kg).
From day 4 to day 13 non-significant differences

GSK'963 (2 mg/Kg) → L19-mTNF (250 µg/Kg) vs GSK'963 (2 mg/Kg) → L19-mTNF (375 µg/Kg).
From day 4 to day 13 non-significant differences

Body Weight Change (%)

GSK'963 (2 mg/Kg) vs L19-mTNF (250 µg/Kg).
day 4 non-significant differences
day 5 $p < 0.05$
day 6 $p < 0.01$
day 7 $p < 0.0001$
day 8 $p < 0.01$
day 9 $p < 0.01$
From day 10 non-significant differences

GSK'963 (2 mg/Kg) vs GSK'963 (2 mg/Kg) → L19-mTNF (250 µg/Kg).
From day 4 to day 13 non-significant differences

L19-mTNF (250 µg/Kg) vs GSK'963 (2 mg/Kg) → L19-mTNF (250 µg/Kg).
From day 4 to day 13 non-significant differences

GSK'963 (2 mg/Kg) vs L19-mTNF (375 µg/Kg).
day 4 non-significant differences
day 5 $p < 0.001$
day 6 $p < 0.0001$

GSK'963 (2 mg/Kg) vs GSK'963 (2 mg/Kg) → L19-mTNF (375 µg/Kg).
From day 4 to day 6 non-significant differences
day 7 $p < 0.05$
day 8 non-significant differences
day 9 $p < 0.05$
from day 10 non-significant differences

L19-mTNF (375 µg/Kg) vs GSK'963 (2 mg/Kg) → L19-mTNF (375 µg/Kg).
day 4 non-significant differences
day 5 non-significant differences
day 6 $p < 0.05$

L19-mTNF (250 µg/Kg) vs L19-mTNF (375 µg/ Kg).
From day 4 to day 6 non-significant differences

L19-mTNF (250 µg/Kg) vs GSK'963 (2 mg/Kg) → L19-mTNF (375 µg/Kg).
From day 4 to day 13 non-significant differences

GSK'963 (2 mg/Kg) → L19-mTNF (250 µg/Kg) vs GSK'963 (2 mg/Kg) → L19-mTNF (375 µg/Kg).
From day 4 to day 13 non-significant differences

Tumor Size (mg)

Ibuprofen (5 mg/Kg) vs L19-mTNF (375 µg/Kg).
From day 10 to day 14 non-significant differences
day 16 $p < 0.001$

Ibuprofen (5 mg/Kg) vs ibuprofen (5 mg/Kg) → L19-mTNF (375 µg/Kg).
From day 10 to day 15 non-significant differences
day 16 $p < 0.001$

L19-mTNF (375 μg/Kg) vs ibuprofen (5 mg/Kg) → L19-mTNF (375 μg/Kg).

From day 10 to day 16 non-significant differences

Body Weight Change (%)

Ibuprofen (5 mg/Kg) vs L19-mTNF (375 μg/Kg).

From day 10 to day 13 non-significant differences

day 14 $p < 0.001$

from day 15 $p < 0.0001$

Ibuprofen (5 mg/Kg) vs ibuprofen (5 mg/Kg) → L19-mTNF (375 μg/Kg).

From day 10 to day 13 non-significant differences

day 14 $p < 0.001$

from day 15 $p < 0.0001$

L19-mTNF (375 μg/Kg) vs ibuprofen (5 mg/Kg) → L19-mTNF (375 μg/Kg).

From day 10 to day 16 non-significant differences
